# Supplementary material for: Improved Adsorption of the Toxic Herbicide Diuron Using Activated Carbon Obtained from Residual Cassava Biomass (Manihot esculenta)
Source: Molecules. 2022 Nov 4;27(21):7574. doi: 10.3390/molecules27217574 (PMC9656765; doi:10.3390/molecules27217574)

Supplementary material

S1. Activated carbon preparation

First, the husks were washed several times until all soil particles were removed from the material. Then the material was transformed into particles with a size of 5 cm. All the solid was taken to the oven for 48 hours at a temperature of 328.15 K. Then it was crushed in a knife mill, and then the biomass was sieved, generating a powder with a particle size of less than 0.20 mm. A darker-colored powder with a moisture content of 7.5 ± 0.5 % (wet basis) was obtained, called precursor material. About 5g of this material was separated for characterization analysis and called Cassava husk (CH), and the remainder was used to obtain activated carbon.

In this step, 25 g of the precursor material was used with 25 g of $ZnCl_2$ (portions of 1:1) and then mixed with distilled water until a homogeneous dark-colored paste was obtained. The paste was taken to the oven and dried at 378 K for 48 h. Then it was crushed again until particles with a diameter smaller than 355 µm were obtained. Then, the pyrolysis step was carried out using a quartz tube under an N_2 atmosphere with a flow rate of 0.25 L min^{-1}, containing the mixture, under a heating rate of 283.15 K min-1 until the temperature reached 923.15 K, maintained for 80 min at this temperature. After this period, the material obtained by pyrolysis was placed in contact with an HCl solution (10 mol L^{-1}) for 120 min, carrying out the acid extraction (removing all the $ZnCl_2$ present in the sample); in this step, the pH of the solution was measured several times until reaching neutrality (pH=7). Finally, the solid particles were washed with distilled water, separated by decantation, and taken to an oven at 323.15 K for 300 min. After drying, the sample was massed and sieved, obtaining particles with a diameter of less than 149 µm, and entitled Cassava peels activated carbon (CPAC), in which a part was taken to the characterization analysis and the remainder used for the herbicide adsorption studies.

S2. Characterization techniques

Fourier-transform infrared spectroscopy (FT-IR) was performed by an IR-Prestige-21 (Shimadzu) equipment to obtain information regarding the chemical structure of the raw and modified materials. In this step, 50 mg of each material was pressed separately with 50 mg of dry potassium bromide. The spectra were obtained with a resolution of 4 cm^{-1}, at several sequential scans in the range of 4500-500 cm^{-1}. The respective analyzes were carried out at room temperature (298 K) in a dehumidified room. Scanning electron microscopy (SEM) analysis was performed on a Vega 3 SB (Tescan) apparatus, with a working voltage of 10 kV, and image magnification of 500x, 1000x, and 3000x, to investigate the surface morphology of the materials. X-ray diffraction (XRD) analysis was performed with the aid of a computer-controlled X-ray diffractometer (Miniflex 300, Rigaku) to identify the crystallographic structure. The N_2 adsorption/desorption isotherms were performed using an ASAP 2020 (Micrometrics) apparatus. The material's specific surface area (SBET, m^2 g-1) was estimated based on the Brunauer-Emmett-Teller (BET) theory. The total pore volume (V_T, cm^3 g^{-1}) was determined considering the amount of N_2 adsorbed on the material surface at a relative pressure (P/P_0) of 0.99. The total pore volume was determined considering the N_2 volume adsorbed at P/P_0 values of 0.1 and 0.95 using the DFT method. Scanning electron microscopy (SEM) (Vega 3 SB, Tescan) was used to obtain the material surface image and its atomic composition. The estimation of the activated carbon yield (Y, %) according to Equation 1:

$$Y = \left(1 - \frac{m_f}{m_i}\right) \cdot 100\ \% \quad (1)$$

Where m_i is the initial precursor mass (g), m_f is the precursor mass after the pyrolysis

S2. Batch adsorption equations

The adsorption capacity at any time (q_t, mg g^{-1}), adsorption capacity at the equilibrium (q_e, mg g^{-1}), and removal percentage (R, %) values were calculated by the Equations (2), (3) and (4), respectively:

$$q_t = (C_0 - C_t)\,\frac{V}{m} \tag{2}$$

$$q_e = (C_0 - C_e)\,\frac{V}{m} \tag{3}$$

$$R = \frac{(C_0 - C_t)}{C_0}\,100 \tag{4}$$

Where: C_0 is the initial adsorbate concentration in the liquid phase (mg L^{-1}), C_e is the equilibrium concentration of the adsorbate in the liquid phase (mg L^{-1}), C_t is the adsorbate concentration in the liquid phase at any time (mg L^{-1}), m is the mass of adsorbent (g), and V is the volume of solution (L).

S3. Isotherm models

The Langmuir model (Eq. 5), Freundlich model (Eq. 6), and Dubinin–Radushkevich model (Eq. 7) are presented as follows:

$$q_e = q_L \frac{K_L C_e}{1 + K_L C_e} \tag{5}$$

$$q_e = K_F C_e^{1/n_f} \tag{6}$$

$$q_e = q_{mDR} \exp\left(-\beta \varepsilon^2\right) \tag{7}$$

$$\varepsilon = RT\ln\left(\frac{C_S}{C_e}\right) \tag{7.a}$$

Where: q_L is the maximum adsorption capacity of Langmuir model (mg g^{-1}), K_L is the Langmuir constant (L mg^{-1}); K_F is the Freundlich constant ((mg g^{-1}) (mg $L^{-1})^{-1/n}$); $1/n_f$ (dimensionless) is the heterogeneity factor; β is the Dubinin–Radushkevich constant (mol^2 kJ^{-2}); R is the universal gas constant (8,314 J mol^{-1} K^{-1}); C_S is the diuron solubility; T is the temperature (K).

S4. Thermodynamics estimation

The Gibbs free energy (ΔG^0, kJ mol^{-1}), enthalpy change (ΔH^0, kJ mol^{-1}), and entropy change (ΔS^0, kJ mol^{-1} K^{-1}) were calculated by the following Equations:

$$K_e = \frac{K_F \varrho}{1000}\left(\frac{10^6}{\varrho}\right)^{\left(1-\frac{1}{n_f}\right)} \tag{8}$$

$$\Delta G^0 = -RT\ln(K_e) \tag{9}$$

$$\Delta G^0 = \Delta H^0 - T\Delta S^0 \tag{10}$$

$$\ln(K_e) = \frac{\Delta S^0}{R} - \frac{\Delta H^0}{RT} \tag{11}$$

Where: K_e is the equilibrium constant (dimensionless); K_F is the Freundlich constant the constant ((mg g^{-1}) (mg $L^{-1})^{-1/n}$), /n (dimensionless) is the heterogeneity factor, ϱ is the density of pure water (assumed 1.0 g mL^{-1}), T is the temperature (K); R is the universal gas constant (8.31 × 10^{-3} kJ mol^{-1} K^{-1}).

S5. LDF model

For describing the adsorption kinetics, the linear driving force model (LDF) was selected, where the adsorption rate can be expressed as:

$$\frac{dq_t}{dt}=k_{LDF}\left(q^*-q_t\right) \tag{12}$$

$$q_t\ (t=0)=0 \tag{12.a}$$

where: q_t is the adsorption capacity at any time (mg g^{-1}), q^* is the adsorption capacity at the equilibrium (mg g^{-1}), k_{LDF} is the LDF mass transfer coefficient (min^{-1}). The solution of the model depends on the best-fitted isotherm, and in this case, the Freundlich model was chosen:

$$q^*=K_F C_e^{1/n} \tag{13}$$

The equilibrium concentration can be correlated with the adsorption capacity through the mass balance equation:

$$C_e = C_0-D_0\bar{q} \tag{14}$$

Where C_0 is the initial concentration (mg L^{-1}), D_0 is the adsorbent dosage (g L^{-1}).
Substituting Eqs. (13) and (14) into (12), results:

$$\frac{d\bar{q}}{dt}=k_{LDF}\left(K_F\left(C_0-D_0\bar{q}\right)^{1/n}-\bar{q}\right) \tag{15}$$

The diffusivity for a homogeneous particle can be estimated from the LDF kinetic lumped parameter:

$$D_S=\frac{{R_p}^2 k_{LDF}}{15} \tag{16}$$

R_P is the adsorbent particle radius, and D_S is the surface diffusivity (cm^2 s^{-1}).

S6. Statistical evaluation

The determination coefficient (R^2) (Eq. 17), adjusted determination coefficient (R^2_{adj}) (Eq. 18), average relative error (ARE) (Eq. 19), and mean square error (MSR) (Eq. 20) are given by the following Equations:

$$R^2 = 1-\frac{\sum_{i=1}^{n}\left(y_{exp}-y_{pred}\right)^2}{\sum_{i=1}^{n}\left(y_{exp}-\bar{y}_{exp}\right)^2} \tag{17}$$

$$R^2_{adj} = 1-\left(1-R^2\right)\frac{(n-1)}{(n-p-1)} \tag{18}$$

$$ARE = \frac{100\%}{n}\sum_{i=1}^{n}\left|\frac{y_{exp}-y_{pred}}{y_{exp}}\right| \tag{19}$$

$$MSR = \frac{1}{n-p}\sum_{i=1}^{n}\left(y_{exp}-y_{pred}\right)^2 \qquad (20)$$

where: y_{exp} is the experimental value; y_{pred} is the predicted value; n is the number of experimental values; p is the number of parameters according to the model.

S7Supplementary results

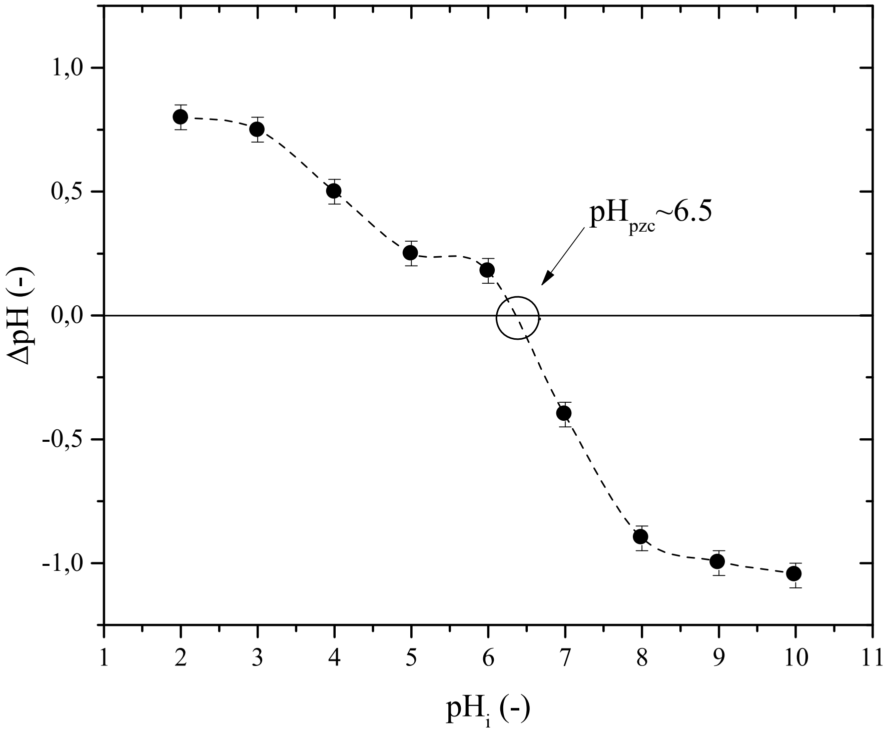

Supplement: Supplementary file 1 [file molecules-27-07574-s001.zip › molecules-1966982-supplementary.pdf]
